# Neural crest cells require Meis2 for patterning the mandibular arch via the Sonic hedgehog pathway

**DOI:** 10.1242/bio.052043

**Published:** 2020-06-25

**Authors:** Jaroslav Fabik, Katarina Kovacova, Zbynek Kozmik, Ondrej Machon

**Affiliations:** 1 Department of Developmental Biology, Institute of Experimental Medicine of the Czech Academy of Sciences, Praha, Czech Republic; 2 Department of Cell Biology, Faculty of Science, Charles University, Praha, Czech Republic; 3 Laboratory of Transcriptional Regulation, Institute of Molecular Genetics of the Czech Academy of Sciences, Praha, Czech Republic

**Keywords:** Meis, Craniofacial, Sonic hedgehog (Shh) signalling, Pharyngeal arch

## Abstract

Cranial neural crest cells (cNCCs) originate in the anterior neural tube and populate pharyngeal arches in which they contribute to formation of bone and cartilage. This cell population also provides molecular signals for the development of tissues of non-neural crest origin, such as the tongue muscles, teeth enamel or gland epithelium. Here we show that the transcription factor Meis2 is expressed in the oral region of the first pharyngeal arch (PA1) and later in the tongue primordium. Conditional inactivation of *Meis2* in cNCCs resulted in loss of Sonic hedgehog signalling in the oropharyngeal epithelium and impaired patterning of PA1 along the lateral–medial and oral–aboral axis. Failure of molecular specification of PA1, illustrated by altered expression of *Hand1/2*, *Dlx5*, *Barx1*, *Gsc* and other markers, led to hypoplastic tongue and ectopic ossification of the mandible. *Meis2*-mutant mice thus display craniofacial defects that are reminiscent of several human syndromes and patients with mutations in the *Meis2* gene.

## Abstract (precis)

**Summary:** Wnt1-Cre2;*Meis2*^fl/fl^ mutants have perturbed Shh activity in the oral region of the mandibular arch, which leads to specification failure of NCCs in the tongue primordium and ectopic ossification at the expense of glossogenesis, which are hallmarks of several mouse mutants with eliminated Shh activity.

## INTRODUCTION

Craniofacial development requires a coordinated integration of various tissues. The vertebrate skull represents a meeting place of two robust mesenchymal populations, the neural crest and cranial mesoderm, both of which make up the skeleton, connective tissues and muscles of the skull and tongue (Noden and Trainor, 2005). The vast majority of the craniofacial skeleton and connective tissues are derived from neural crest cells (NCCs). NCCs are a migratory, multipotent stem cell population that originate from the dorsal neural folds and are capable of differentiating into a plethora of tissue types, including bone, cartilage, neurons and pigment cells (Baggiolini et al., 2015). In the neural tube, NCCs can be divided into four domains along the anterior–posterior axis. The anterior-most population, termed cranial neural crest, has skeletogenic properties and colonises the frontonasal prominence and pharyngeal arches (PAs) where it interacts with adjacent tissues to control the craniofacial morphogenesis.

PAs are a series of bilaterally symmetrical outgrowths on the sides of the developing pharyngeal cavity. In humans and mice, there are five PAs. Cranial neural crest cells (cNCCs) populate PAs in distinct segregated streams. The segmentation and identity of these streams in PAs are defined by the spatiotemporal expression of *Hox* genes in the hindbrain (Parker et al., 2018). Each PA shares a basic structure that is composed of all germ layers – surface and oral epithelium from the ectoderm, pharyngeal epithelium from the endoderm and PA's core of intermingled mesoderm and NCCs.

cNCCs with skeletogenic properties give rise to bone, cartilage and connective tissues of structures derived from PAs (Frisdal and Trainor, 2014). The PA1 cartilage palatoquadratum forms the incus and a part of orbital wall (alisphenoid), while the PA1 cartilage Meckel's forms the malleus. Adjacent cNCCs in the PA1 undergo direct ossification to form the dermal bones of the upper and lower jaw. PA2 cartilage forms the stapes, the styloid process of temporal bone and the lesser horns of the hyoid bone. Greater horns and the body of hyoid bone arise from the PA3 cartilage, while PA4 forms thyroid cartilage (Tabler et al., 2017). Alongside bone and cartilage, all PAs contribute to the formation of tongue tissues (Cobourne et al., 2019). The oral part of the tongue originates from PA1, while the pharyngeal part derives from the PA3 and PA4. Differentiation of mesenchymal cells in PAs depends on environmental cues they receive from the adjacent epithelia. To organise bone and tongue formation in the PA1, the oral epithelium interacts with underlying cNCC-derived mesenchyme. Upon initiation of gross development of the tongue, three elevations emerge on the surface of the mandibular prominence. They make contact in the midline, fuse and form a tongue primordium. A midline elevation, derived from the PA3 and PA4, arises at the posterior aspect of the pharyngeal cavity and fuses with the tongue primordium to create the pharyngeal part of the tongue.

The early signals driving cNCC-derived mesenchyme into a tongue lineage involve major signalling pathways. Bone morphogenetic protein (Bmp) signalling emanating from the oral ectoderm acts to divide the nascent mandible into a nested subdomain characterised by the expression of Dlx homeobox and Hand basic helix-loop-helix transcription factors (Charité et al., 2001; Depew et al., 2002, 2005; Medeiros and Crump, 2012; Vincentz et al., 2016). While the expression of *Hand1* is induced by Bmp signalling itself, *Hand2* expression requires the presence of Dlx5/6-signalling proteins in the arch (Vincentz et al., 2016). These signalling proteins upregulate the expression of *Hand2*, which in return activates the expression of Hand1. *Hand2* expression synergistically acts with Bmp to regulate the expression of *Hand1* (Barron et al., 2011; Vincentz et al., 2016). However, *Hand1* expression is inhibited by Dlx5/6, meaning that the *Hand2* reduction results in marked reduction of *Hand1* in the arch. More importantly, *Hand2* plays a major role in establishing a negative-feedback loop in Dlx5/6-Runx2 circuit. Altogether, the nested expression of Dlx and Hand genes in the mandibular arch is a vital step in the formation of jaw-specific structures, including heterogeneous teeth, bone and tongue.

Sonic hedgehog (Shh) is expressed in the epithelium of an early oropharynx, where it acts as a signalling centre for development of oral structures, including the tongue, teeth, palate, and salivary glands. At embryonic day (E) 9.5 in mouse, Shh is expressed in the epithelial lining of PA1, even prior to the formation of tongue primordium, and determines tongue and mandible morphogenesis. Later on, the expression localises to the lateral–distal epithelium of tongue primordium and then to nascent tongue papillae as tongue development proceeds (Jung et al., 1999). Both epithelial and mesenchymal cells of PA1 express receptors *Smo* and Ptch1 and thereby respond to Shh ligand and transduce Shh signalling via a primary cilium and transcription factors of the Gli family. Elimination of epithelial either *Shh* or *Smo* in NCCs leads to failure of patterning of PA1, abrogation of tongue development and truncation of the mandible (Billmyre and Klingensmith, 2015; Jeong et al., 2004; Xu et al., 2019). Similar findings were reported after genetic removal of primary cilia in NCCs or in mandible explants after blocking Shh *in vitro* (Liu et al., 2004; Millington et al., 2017). Shh thus exerts numerous functions during tongue development and has been linked to the survival of the NC-derived mesenchyme and mesodermal myogenic progenitor cells (Jeong et al., 2004; Millington et al., 2017). Intriguingly, it has been recently reported that Shh is involved in the oral–aboral patterning of the mandibular arch via restricting Bmp signalling to the aboral region of PA1. Ablation of *Smo* in the NC-derived mesenchyme led to a mirror-image duplication of mandibular bone in the oral region, showing that Shh–Bmp complementary gradients define the patterning of oral–aboral axis of the nascent mandible (Xu et al., 2019).

Meis2 is a transcription factor that plays multiple roles in development and cancer. It is involved in embryonic development of numerous organs, including the heart, pancreas, eye lens, brain and neural crest (Agoston et al., 2014; Antosova et al., 2016; Conte et al., 2010; Machon et al., 2015; Wu et al., 2015; Zhang et al., 2006). Its DNA-binding homeodomain contains a three-amino-acid loop extension (TALE subclass). Transcription factors of the Meis family directly bind to Pbx proteins and Meis/Pbx protein complexes bind to a DNA via respective Meis- and Pbx-consensus binding sites (Schulte and Geerts, 2019). In humans and mice, three paralogues of the Meis family have been identified. Recently, several patients with congenital craniofacial malformations such as cleft palate have been described as carrying heterozygous mutations in *MEIS2* gene (Crowley et al., 2010; Douglas et al., 2018; Erdogan et al., 2007; Giliberti et al., 2019; Johansson et al., 2014; Verheije et al., 2019). These craniofacial abnormalities were often co-occurring with cardiac septal defects, gastroesophageal reflux disease and intellectual disability. Patients also presented with recurrent dysmorphic facial features that delineated a distinct MEIS2-mutation specific facial phenotype. Worthy of note, a subset of patients afflicted with MEIS2 haploinsufficiency also presented with jaw anomalies, e.g. retrognathia, micrognathia, microstomia and dental anomalies (Chen et al., 2016; Douglas et al., 2018; Erdogan et al., 2007; Verheije et al., 2019) that relate to the prenatal development of the mandibular prominence. Moreover, haploinsufficiency of MEIS2 is occasionally reported in patients with 15q14 microdeletion syndrome and expanded Prader–Willi syndrome, where loss of single MEIS2 copy has been linked to the more severe clinical presentation of the phenotype. (Liu et al., 2013). According to some authors, the *MEIS2* gene should be considered among the candidate causative genes in cases without 22q11.2 deletions in patients with cleft palate (Johansson et al., 2014). Altogether, haploinsufficiency of Meis2 could present as a standalone clinical entity or as an additional component of broader syndromic diseases (Liu et al., 2013; Roberti et al., 2011; Shimojima et al., 2017, 14). We have previously reported that both systemic and conditional inactivation of *Meis2* during mouse embryonic development resulted in craniofacial and cardiac defects (Machon et al., 2015). In this paper, we specifically focus on the function of *Meis2* during development of the mandibular arch in the mouse. Using Wnt1-Cre2-faciliated genetic ablation of *Meis2* in NCCs we show that Meis2 acts upstream of Shh signalling during the patterning of PA1 and is critical for morphogenesis of the tongue and mandible.

## RESULTS

### *Meis2* deletion leads to hypoglossia and ectopic ossification in the mandible

**Fig. 1.**
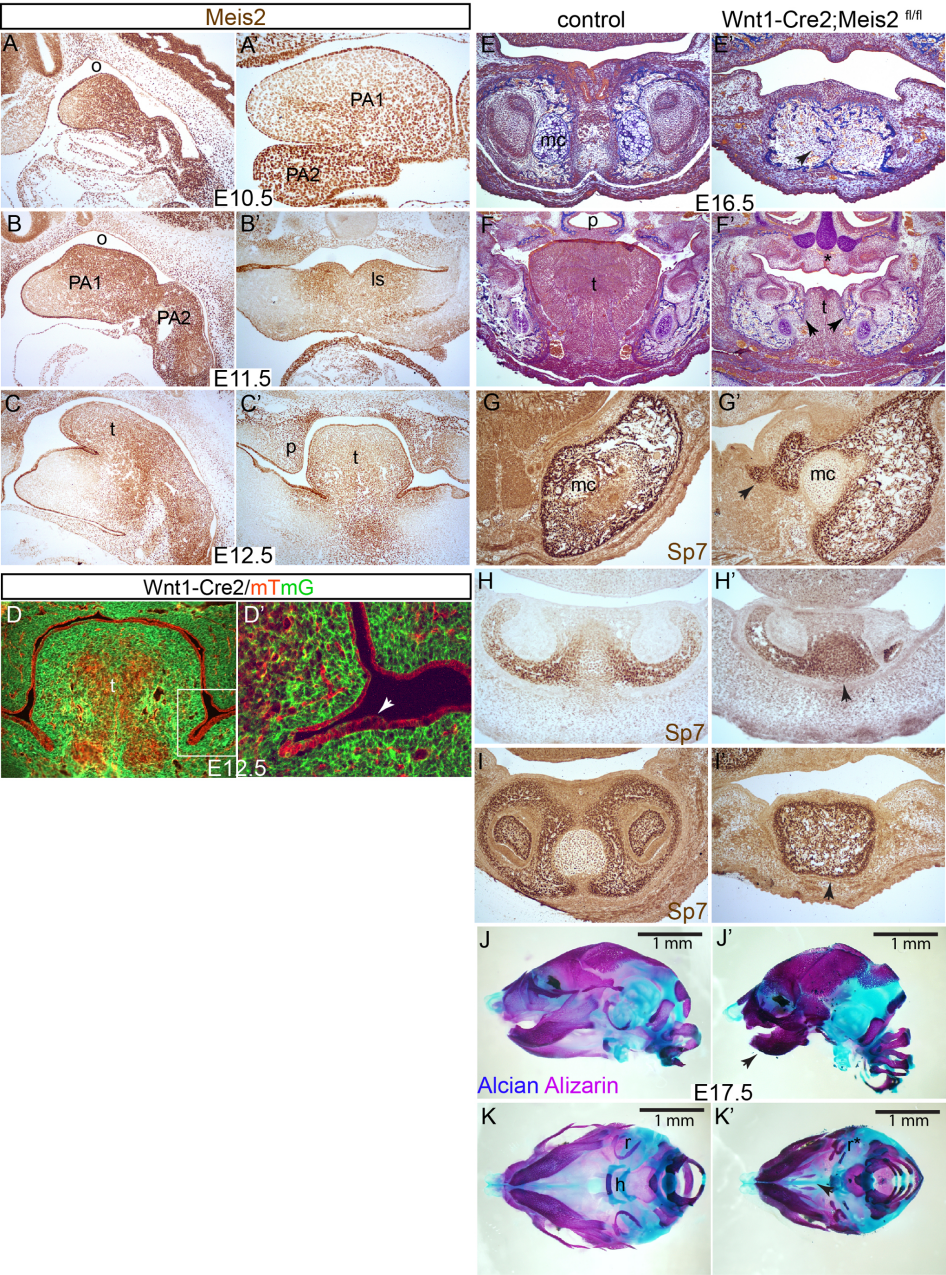
**Conditional deletion of Meis2 results in tongue hypoplasia and impaired mandible development.** (A–C’) Meis2 immunohistochemistry between E10.5–E12.5 showing Meis2 expression in pharyngeal arches and tongue primordium, (A–C) sagittal sections, (A’–C’) frontal sections. (D,D’) Lineage tracing of Wnt1-Cre2 at E12.5 using mTmG mouse strain that was used for inactivation of *Meis2*^fl/fl^. Wnt1-Cre2 is active in the mesenchyme of NC origin (GFP, green), whereas the oral ectodermal epithelium and mesodermal myogenic progenitors do not undergo Cre recombination (tdTomato, red), D’ shows magnified area depicted in D. Note that oral epithelial cells were not targeted by Wnt1-Cre2 (arrow), frontal sections. (E,E’) Trichrome staining at E16.5. Note the distal mandible showing abnormal fusion of the distal tip (arrow) and aberrant ossification in Wnt1-Cre2;*Meis2* ^fl/fl^ embryos, frontal sections. (F–F’) Trichrome staining at E16.5. The molar region in mutants shows severe hypoplasia of tongue, cleft palate (*) and aberrant ossification around the lingual grooves (arrows), frontal sections (G,G’) Sp7 immunohistochemistry at E16.5 showing aberrant ossification in the medial region close to tongue and around lingual groove (arrow), frontal sections. (H,H’) Sp7 immunohistochemistry at E14.5 of the distal mandible showing ectopic ossification of Meckel's cartilage (arrow), frontal sections. (I,I’) Sp7 immunohistochemistry at E16.5 showing abnormal fusion of the distal tip of mandible (arrow), frontal sections (J–K’) Alcian and Alizarin staining of bone and cartilage at E17.5. Note the micrognathia in the mutant (J’, arrow); lateral (J,J’) and ventral views (K,K’). ls, lingual swellings; mc, Meckel's cartilage; o, oral side; p, palate; r, tympanic rings; t, tongue.

Our previous work has documented that Meis2 transcription factor is abundantly expressed in cranial neural crest cells and is necessary for osteochondrogenic differentiation in the developing mandible as well as in other bones and cartilages originating from PAs (Machon et al., 2015). To get better insight into the molecular mechanism leading to severe craniofacial defects in *Meis2*-deficient mice, we generated conditional mutants employing Wnt1-Cre2 mouse strain that is widely used for recombination in NCCs (Lewis et al., 2013). As mouse *Meis1* and *Meis2* paralogues are structurally very similar and their homeodomains almost identical, we wanted to verify a potential functional redundancy of both paralogues during NCC development. We therefore crossed floxed alleles *Meis1*^fl/fl^ and *Meis2*^fl/fl^ to obtain embryos lacking either *Meis1* or *Meis2*. Embryos were harvested at 15 days post coitum (E15.5) and gross morphology was examined using computed microtomography scanning (micro CT). Fig. S1 shows that Wnt1-Cre2;*Meis1*^fl/+^;*Meis2*^fl/fl^ mutants exhibited cleft palate, underdeveloped tongue (hypoglossia) and small mandible (micrognathia). In contrast, Wnt1-Cre2;*Meis1*^fl/fl^;*Meis2*^fl/+^ embryos appeared normal in comparison to control littermates. We conclude that craniofacial morphogenesis is controlled by Meis2-dependent NCC development while Meis1 seems dispensable for craniofacial morphogenesis in our experiments. In the following analyses, we focused only on Meis2 conditional mutants. At first, we carefully mapped the expression pattern of Meis2 during critical stages of PA1 development between E10-E12.5 using immunohistochemistry. As shown in Fig. 1A–C’, Meis2 protein is abundant in PA1 and PA2 showing graded expression with a stronger signal on the oral side (o) of PA1 and in the tongue region of PA1. Particularly around the midline region of PA1, lingual swellings (ls) at E11.5 (Fig. 1B’), and later tongue primordium (t) at E12.5 (Fig. 1C,C’), display the strongest signal. Wnt1-Cre2-mediated recombination was mapped after crossing to the reporter strain mTmG in which GFP fluorescence is activated in cells after recombination (green) while cells without Cre maintained tdTomato expression (red). In the developing tongue at E12.5, the majority of cells were found to be of NCC origin while two tdTomato-positive zones in the midline contain the population of mesodermal myogenic progenitors that have migrated into the tongue (Fig. 1D). Oral epithelial cells were not targeted by Wnt1-Cre2 (Fig. 1D’, arrow).

Next, we performed Mallory's trichrome histological staining of frontal sections of Wnt1-Cre2;*Meis2*^fl/fl^ at E16.5. In these conditional mutants, we observed ectopic ossification and fusion of the mandibular bone in the distal region (Fig. 1E’, arrow). Moreover, the tongue (t) in the molar region was almost absent (Fig. 1F’). The lingual epithelium structure seemed impaired. Palatal shelves were hypoplastic and formation of the secondary palate (p) was abrogated (asterisk in Fig. 1F’). We also observed ectopic ossification in the area near the tongue, particularly around the lingual groove, an epithelial invagination separating tongue and future tooth-bearing alveolar bone (Fig. 1F’, arrows). To validate this, we used immunohistochemical staining of Sp7 that specifically labels bone-matrix secreting osteoblasts. Indeed, ectopic expression of Sp7 was detected medially to Meckel's cartilage (mc) in conditional mutants at E16.5 (arrow in Fig. 1G’). Ectopic expression of Sp7 was also observed in the distal tip of mc at E14.5 (arrow in Fig. 1H’), which, in some cases, may have resulted in fusion of the distal mandible (arrow in Fig. 1I’).

To obtain an overall picture of bone and cartilage formation in embryonic heads, Alizarin/Alcian staining was carried out (Fig. 1J–K’). Subsequent analysis of E17 embryos further confirmed anomalies in mandibular development. The mandible is clearly smaller than in control littermates (arrow in Fig. 1J’) and Alizarin staining showed increased staining of calcium, suggesting abnormal ossification. Furthermore, we observed cleft palate (arrow in Fig. 1K’) and malformed tympanic rings (r*). In summary, we conclude that Meis2, but not Meis1, is required for NCCs differentiation in PA1 as well as for the development of several derived structures including the tongue and mandible.

### Decreased Shh activity in Wnt1-Cre2;*Meis2*^fl/fl^ mutants

**Fig. 2.**
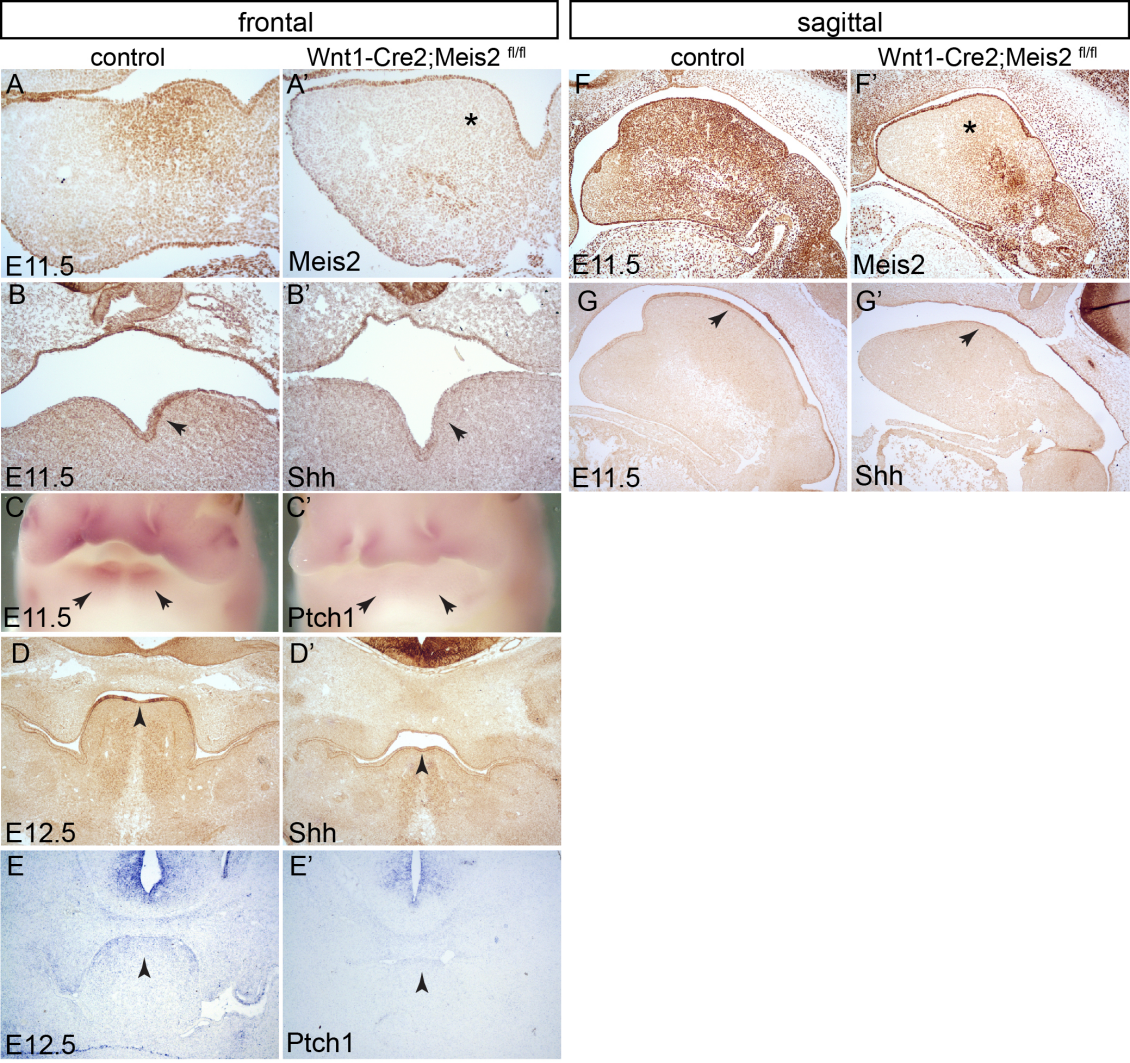
**Meis2 deletion leads to downregulation of Shh signalling in PA1.** (A,A’) Meis2 immunohistochemistry at E11.5. In Wnt1-Cre2;*Meis2* ^fl/fl^ mutants the expression of Meis2 is efficiently removed in the PA1 mesenchyme. *, frontal sections. (B,B’) Shh immunohistochemistry at E11.5 of PA1 illustrates the loss of epithelial expression of Shh in mutants, frontal sections (arrows). (C,C’) *Ptch1* whole-mount *in situ* hybridization at E10.5. *Ptch1* expression disappeared from lingual swellings (arrows) frontal views. (D,D’). Shh immunohistochemistry at E11.5. Tongue primordium exhibits the loss of epithelial expression of *Shh* in mutants (arrows), frontal sections. (E,E’) *Ptch1 in situ* hybridisation at E10.5 showing the loss of *Ptch1* expression in mutant tongue (arrows), frontal sections. (F,F’) Meis2 immunohistochemistry at E11.5 showing deletion of Meis2 in the PA1 mesenchyme (*) in Wnt1-Cre2;*Meis2* ^fl/fl^ mutants, sagittal sections. (G,G’) Shh immunohistochemistry at E11.5. Note the loss of epithelial Shh in the mutants (arrows), sagittal sections. Magnification: 200x (A,B), 20x (C), 100x (D–G).

The first morphological signs of tongue development, termed lingual swellings, emerge in three elevations at E11.0 in the oral region of PA1. This midline area is abundant in Meis2 protein (Fig. 2A,F). Fig. 2A’,F’ illustrate that Meis2 was effectively deleted in NCCs located in PA1 mesenchyme (*) whereas Meis2 presence was maintained in PA1 ectodermal epithelium in Wnt1-Cre2;*Meis2*^fl/fl^ mutants. We further noticed that Shh expression in the oral epithelium is strongest in the tongue primordium which simultaneously expresses a high amount of Meis2 both in the epithelium and in the underlying NCC mesenchyme (Fig. 2A,B,D,F,G). In contrast, in Wnt1-Cre2;*Meis2*^fl/fl^ mutants, the epithelial Shh expression disappeared as seen in frontal and sagittal sections at stages E11.5–E12.5 (Fig. 2B’,D’,G’, arrows). This indicates that Shh signalling is compromised in the absence of Meis2. To verify this, we looked at the expression of *Ptch1*, a gene regulated by Shh activity. Indeed, *Ptch1* mRNA transcripts, detected by *in situ* hybridization, were downregulated in lingual swellings at E11.5 (Fig. 2C–C’, arrows) and also in the tongue primordium at E12.5 (Fig. 2E,E’). Thus, downregulation of *Ptch1* corresponds to the loss of Shh signalling. Our data suggest that Meis2 transcription factor regulates Shh signalling in PA1.

### Mandibular arch patterning along the medial–distal axis is altered in Wnt1-Cre2;*Meis2*^fl/fl^ mutants

**Fig. 3.**
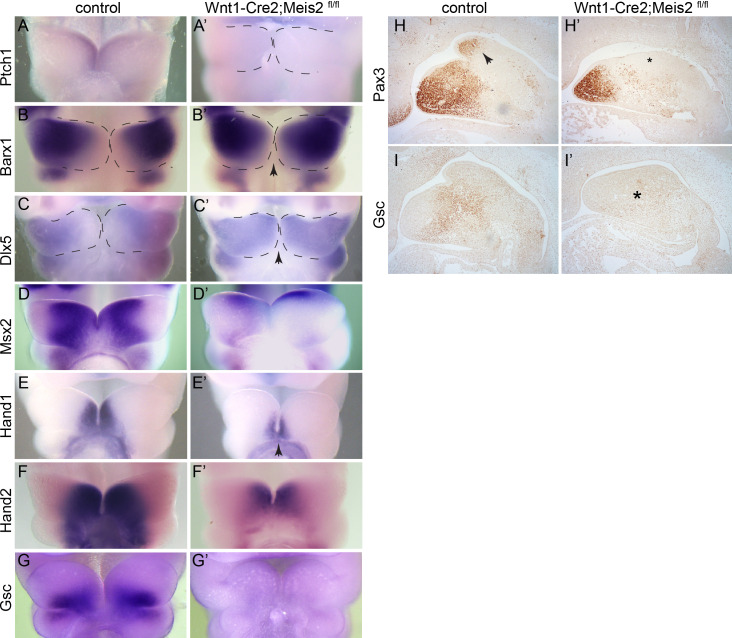
**Molecular patterning of PA1 is impaired in the absence of Meis2.** Edges of PA1 are marked with dashed lines. (A,A’) *Ptch1* whole-mount *in situ* hybridization at E10.5. *Ptch1* expression was lost in Wnt1-Cre2;*Meis2* ^fl/fl^ mutants, frontal views. (B,B’) *Barx1* expression at E10.5 shifted to medial–distal tip of PA1 in mutants (arrow), frontal views. (C,C’) *Dlx5* expression at E10.5 shifted to medial–distal tip of PA1 in mutants (arrow), frontal views. (D,D’) *Msx2* expression at E10.5 almost disappeared in mutants, frontal views. (E–F’) *Hand1* and *Hand2* expression at E10.5 were downregulated in medial–distal tips of PA1 in mutants (arrow), frontal view. (G,G’) *Gsc* expression at E10.5 was lost in mutants; frontal views. (H,H’) Pax3 immunohistochemistry at E11.5. Note a complete loss of Pax3 (*) in the distal tip (arrow) of emerging tongue, sagittal sections. (I,I’) Gsc immunohistochemistry at E11.5, sagittal sections. Magnification: 40× (A–G), 100× (H,I).

Recently, it has been shown that Shh signalling in the oral ectodermal epithelium controls molecular patterning of PA1 (Xu et al., 2019). Oral–aboral, lateral–medial and proximal–distal axes of PA1 are already established at E10 by specific expression of components of Shh, Fgf8 and Bmp4 pathways, namely *Barx1*, *Msx1/2*, *Dlx5/6*, *Ptch1*, *Gsc* or *FoxF1/2*. We therefore tested the effect of *Meis2* deletion on molecular patterning of PA1. *Ptch1*, a downstream target of Shh, was expressed in the medial and mid-oral region of PA1 thus reflecting Shh signalling. In *Meis2* conditional mutants, however, *Ptch1* expression already disappeared at E10.5 (Fig. 3A,A’), which correlates with our findings at E11.5–E12.5 shown above (Fig. 2C,C’–E,E’). Alongside, the expression of *Barx1* and *Dlx5* expanded from proximo-lateral regions towards medio-distal tip of mandibular arch (Fig. 3B–C’, arrow). On the other hand, the expression of *Hand1* and *Hand2*, markers of medial regions of PA1, decreased significantly in Wnt1-Cre2;*Meis2*^fl/fl^ mutants (Fig. 3E–F’, arrow). *Gsc* mRNA was not detected at all in the aboral region of PA1 in mutants at E10.5 (Fig. 3G,G’). This was further validated by immunohistochemical staining of Gsc protein at E11.5 in which the core region of the mutant PA1 lost *Gsc* expression (* in Fig. 3I’). The distal tip of the emerging tongue primordium expresses the transcription factor *Pax3*. Immunohistochemical staining of sagittal sections at E11.5 revealed that the tongue primordium did not bulge out of PA1 in Wnt1-Cre2;*Meis2*^fl/fl^ mutants and Pax3 was not detected in there (Fig. 3H,H’, arrow and *). In conclusion, Wnt1-Cre2;*Meis2*^fl/fl^ mutants exhibit striking differences in the molecular pattern of PA1. Lateral characteristics shifted medially and the medial molecular imprint was strongly reduced. Moreover, both the lateral–medial and oral–aboral axes of PA1 were compromised at E10.5.

### Fgf8 and Bmp pathways are not affected in Wnt1-Cre2;*Meis2*^fl/fl^ mutants

**Fig. 4.**
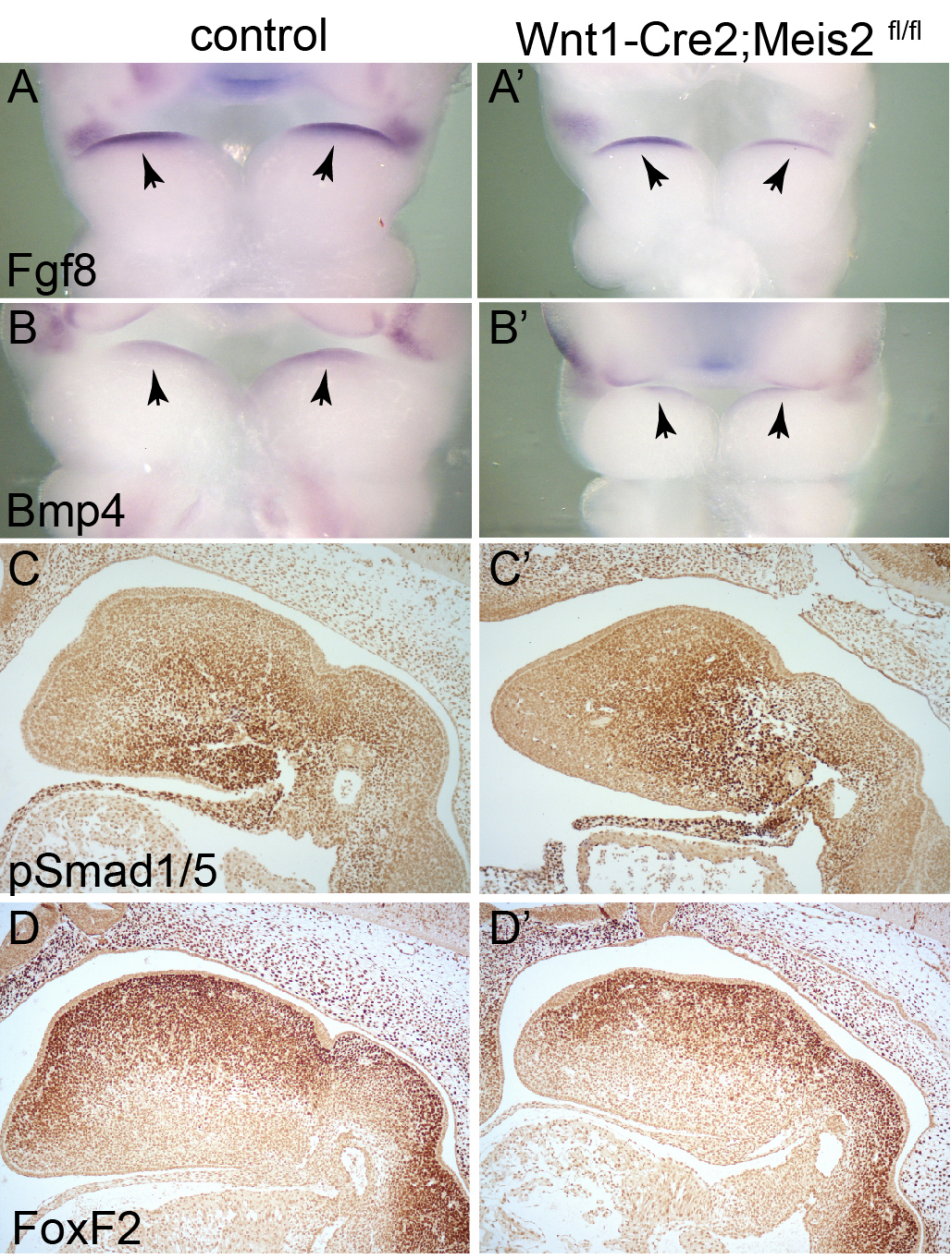
**Fgf and Bmp signalling in PA1 are not affected in Wnt1-Cre2;*Meis2*^fl/fl^ mutants.** (A,A’) *Fgf8* whole-mount *in situ* hybridization at E10.5 in PA1. Note the expression on the oral side (arrows), frontal views. (B,B’) *Bmp4* whole-mount *in situ* hybridization at E10.5 in PA1. Note the expression on the oral side; frontal views (arrows). (C,C’) Phosphorylated Smad1/5 immunohistochemistry at E11.5, sagittal sections (D,D’) FoxF2 immunohistochemistry at E11.5, sagittal sections. Magnification: 50× (A,B), 100× (C,D).

Both Fgf8 and Bmp signalling pathways have been reported to control molecular patterning of PA1 along the proximo–distal and oral–aboral axis (Tucker et al., 1998, 1999; Xu et al., 2019). As many patterning genes are altered in PA1 at E10.5 (see Fig. 3) we examined *Fgf8* and *Bmp4* activity in PA1 in the absence of Meis2. *In situ* hybridization of *Fgf8* and *Bmp4* showed that neither *Fgf8* nor *Bmp4* signal on the oral side of PA1 was changed in Wnt1-Cre2;*Meis2*^fl/fl^ (Fig. 4A–B’). Moreover, expression of phosphorylated Smad1/5, produced upon Bmp pathway activation, was not changed as seen using immunostaining on sagittal sections at E11.5 (Fig. 4C,C’). We also tested expression pattern of FoxF2, a downstream target of Shh activity (Jeong et al., 2004), using immunohistochemistry. Sagittal views at PA1 at E11.5, however, did not show any apparent change in the oral–aboral gradient of FoxF2 in the mutants (Fig. 4D,D’), nor did we detect any change in FoxF1 (data not shown).

### Elevated cell apoptosis in the mandibular arch after elimination of Meis2

**Fig. 5.**
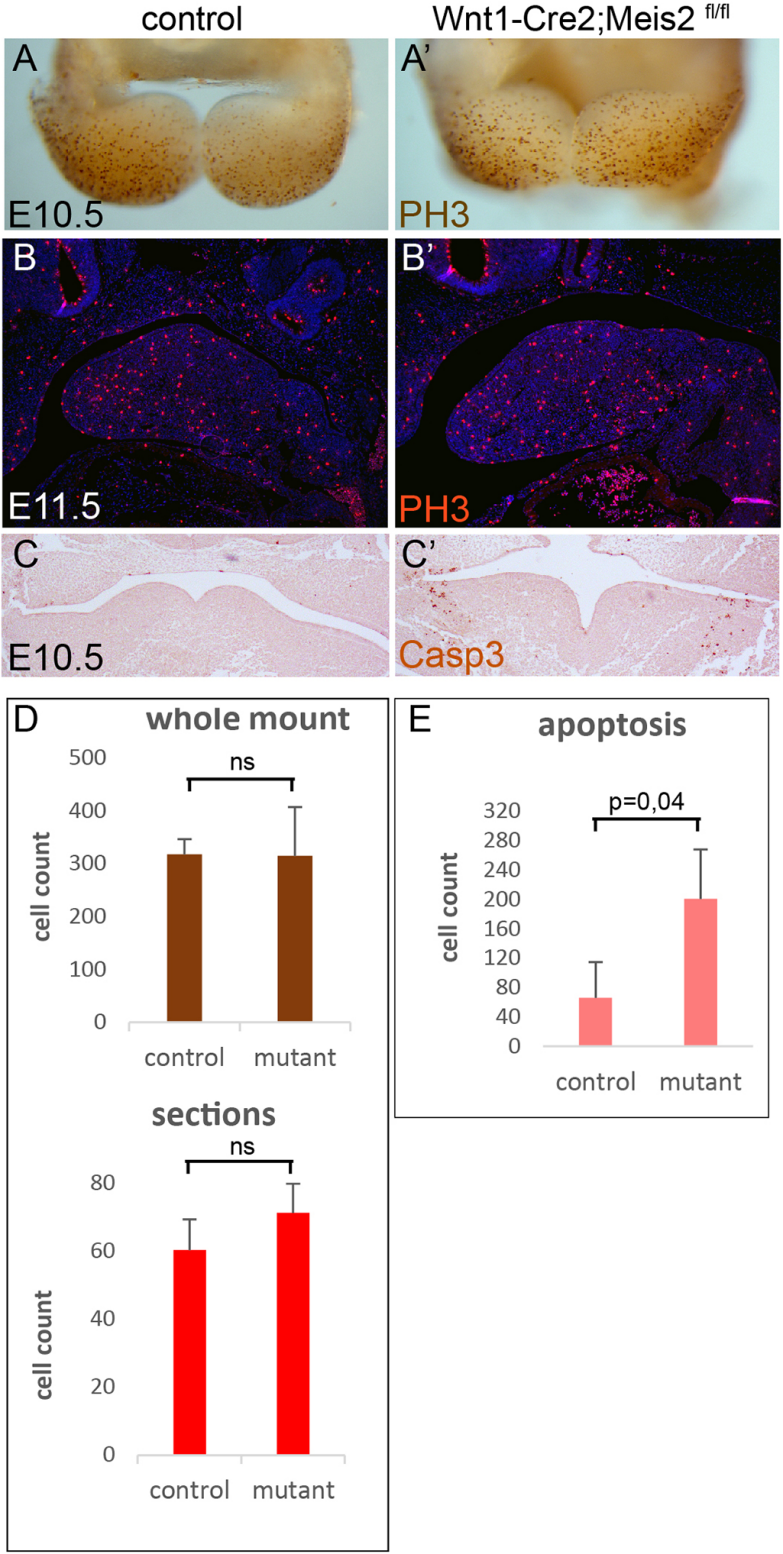
**Increased apoptosis in the mandibular arch (MA) of *Meis2* mutants.** (A,A’) Phospho-histone-3 antibody (PH3) whole-mount staining (brown) at E10.5 that visualises proliferating cells in the MA, ventral views. (B,B’) PH3 immunohistochemistry at E11.5 that visualises proliferating cells in the MA (red), sagittal sections. (C,C’) Caspase-3 (Casp3) immunohistochemistry at E11.5 that visualises apoptotic cells in the MA, frontal sections. (D) Quantification of cell proliferation in the MA in control littermates and mutants. (E) Quantification of apoptotic cells in the MA in control littermates and mutants. Statistical analysis: two mutants and four controls from two independent experiments, three sections for each specimen. ns, not significant; *P*>0.05. Magnification: 40× (A), 100× (B), 200× (C)

Growth retardation of the tongue primordium seen in the Fig. 3H,H’ may be caused by decreased proliferation of NCCs that compose the mandibular arch at early stages. We examined cell proliferation in the conditional mutants using PH3 antibody. Whole-mount immunohistochemistry at E10.5 showed that the overall number of dividing cells labelled with PH3 antibody was not changed in mutants compared to control littermates (Fig. 5A,A’). The number of dividing cells was also counted in sagittal sections at E11.5 (Fig. 5B,B’). Again, we did not measure significant differences in PH3-positive cells between mutants and controls. Quantifications are summarised in Fig. 5D showing average values with standard deviations from three experiments. Next, apoptosis was analysed using Casp3 immunostaining. We detected many apoptotic cells in the mutant PA1 both in frontal sections (Fig. 5C,C’) and in sagittal sections (not shown). Quantification of the level of apoptosis is summarised in Fig. 4E.

### Altered specification of neural crest cells in the tongue primordium

**Fig. 6.**
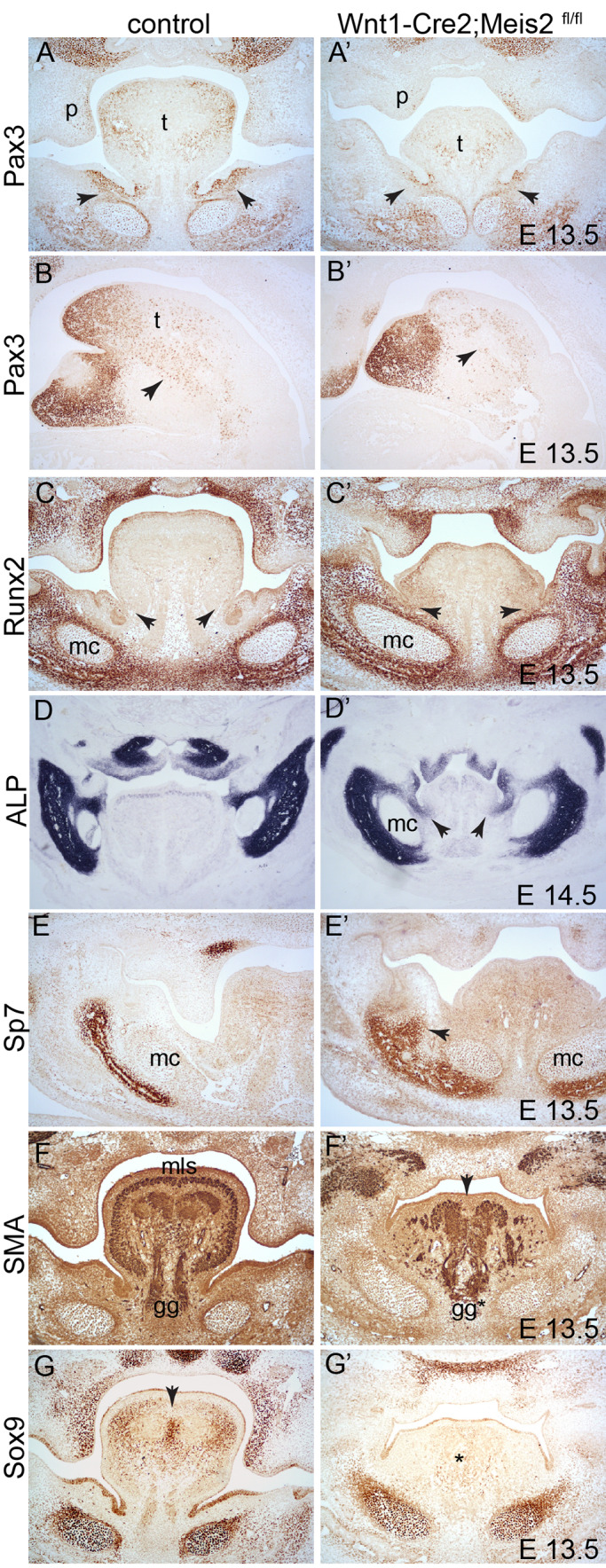
**NCCs in the tongue region differentiate abnormally in Wnt1-Cre2;*Meis2*^fl/fl^ mutants.** (A,A’) Pax3 immunohistochemistry at E13.5; frontal sections. Note decreased expression in the tongue as well as in the mesenchyme around lingual grooves (arrows). (B,B’) Pax3 immunohistochemistry at E13.5. Note loss of *Pax3* expression in the tongue tip and reduced number of Pax3-expressing myogenic progenitors that invade the tongue (arrows), sagittal sections. (C,C’) Runx2 immunohistochemistry at E13.5. Note increased expression in the tongue and around lingual grooves (arrows), frontal sections. (D,D’) ALP staining of active osteoblasts at E14.5 shows abnormal position of ALP-positive cells in proximity of lingual grooves (arrows), frontal sections. (E,E’) Sp7 immunohistochemistry at E16.5. Note ectopically expanded area of Sp7 expression in mutants (arrow), frontal sections. (F,F’) SMA immunohistochemistry at E13.5. Note disorganised pattern of muscle fibres, their reduced number in the tongue and loss of the midline structure (arrow), frontal sections. (G,G’) Sox9 immunohistochemistry at E13.5. Note the loss of Sox9 protein in the tongue (*), especially in the midline (arrow), frontal sections. gg, genioglossus; mc, Meckel's cartilage; mls, musculus longitudinalis superior; p, palatal shelf; t, tongue, magnification: 100× (A-G).

Downregulation of Pax3 in the tongue was also observed in frontal sections at E13.5 (Fig. 6A,A’). However, at this stage, profound morphological changes were apparent, and differences in the distribution of cellular markers may just reflect morphological abnormalities. We observed hypoplasia of palatal shelves (p) (Fig. 6A,A’) and the size of tongue was significantly reduced with lower *Pax3* expression. Remarkably, the mesenchyme around the lingual groove almost lost *Pax3* expression (Fig. 6A’, arrows). The number of myogenic progenitors invading the tongue and expressing moderate levels of Pax3 also appeared lower in sagittal sections in Wnt1-Cre2;*Meis2*^fl/fl^ mutants (Fig. 6B,B’, arrows) which may explain the smaller size of tongue. Reduced Pax3 expression in the mesenchyme surrounding the lingual groove was accompanied with the expansion of Runx2 medially towards the tongue (Fig. 6C,C’, arrows). Elevated Runx2 expression was also observed inside the tongue primordium in mutants whereas the tongue in control littermates was almost devoid of Runx2. Abnormal expression of ossification markers, such as Runx2, in the tongue suggests that NCCs-derived tongue mesenchyme did not follow the correct developmental program and rather adopted the differentiation pathway typical of osteoblast lineage. This hypothesis was further verified by staining alkaline phosphatase (ALP), which is typical of differentiating osteoblasts. As seen in frontal sections at E14.5, ectopic ALP activity was detected around the lingual grooves medially to Meckel's cartilage (Fig. 6D,D’, arrows). We further followed abnormal ossification using immunohistochemistry of Sp7 (a gene downstream of *Runx2*), which, at E13.5, is normally expressed laterally to Meckel's cartilage where the mandibular bone starts forming. In Wnt1-Cre2;*Meis2*^fl/fl^ mutants, however, Sp7 staining expanded superiorly and medially towards lingual groove (Fig. 6E,E’) although the Sp7-positive osteoblast never reached the tongue mesenchyme as was seen in Runx2 stained samples. Next, we examined differentiation of myogenic progenitors into muscle fibres in the tongue using smooth muscle actin (SMA) immunohistochemistry. The number of myogenic progenitors in the tongue was lower and muscle fibres were disarranged in mutants (Fig. 6F,F’, arrow). This suggests that compromised differentiation of NCC, reflected in the ectopic presence of ossification markers in the tongue primordium, leads to inefficient invasion of myogenic progenitors of mesoderm origin into the tongue region.

Tendons of tongue muscles originate from tenocytes, which in the head are derivatives of NCCs. The transcription factor *Sox9* is expressed during chondrocyte, ligament and tenocyte differentiation. In tongue primordium, Sox9 protein is found in lateral and dorsal regions, but also in the midline marking the prospective tendinous lingual septum (Fig. 6G, arrow). In Wnt1-Cre2;*Meis2*^fl/fl^ mutants, however, *Sox9* expression was almost lost both in the midline tendon and in lateral areas (Fig. 6G’, *). This again confirms that NCC differentiation in the tongue primordium is severely affected in the absence of Meis2.

## DISCUSSION

### MA patterning

Our data show that Meis2 regulates Shh expression in the oral epithelium and its loss leads to impaired development of the tongue and mandible. Our findings complement previous reports showing that the elimination of Shh in the oropharyngeal epithelium prior to the formation of the tongue using Nkx2.5-Cre strain causes complete aglossia a micrognathia (Billmyre and Klingensmith, 2015). Wnt1-Cre-mediated deletion of *Smo* in the NCC mesenchyme yields similar morphological abnormalities, i.e. absent tongue and truncated mandible (Jeong et al., 2004; Xu et al., 2019). Alongside aglossia, Wnt1-Cre;Smo^c/c^ mutants develop a mirror-image duplication of mandibular bone. In Wnt1-Cre2;*Meis2*^fl/fl^ mutants, we observed loss of the epithelial Shh ligand in the mandibular arch (MA) that was accompanied by downregulation of Ptch1 receptor. Nonetheless, in comparison with Shh pathway mutants, *Meis2* mutants exhibit milder phenotypes as the tongue is hypoplastic and the mandible is ectopically ossified and truncated. Altogether, this indicates that Shh signalling in the MA is controlled by more complex molecular machinery in which Meis2-mediated transcription takes a major part (Billmyre and Klingensmith, 2015; Jeong et al., 2004; Xu et al., 2019).

*Hand1* and *Hand2* are expressed in medial–distal regions of the MA where they act to specify the distal tip. These transcription factors are downregulated in *Meis2* conditional mutants, but also in Shh ^−/−^ mutants and Wnt1-Cre;Smo^c/c^ mutants (Barron et al., 2011; Jeong et al., 2004; Yamagishi et al., 2006). Transcription factors Dlx5 and Barx1 are expressed in the lateral–proximal regions of the MA, where they orchestrate osteogenesis and odontogenesis under normal conditions. Given the mutually exclusive expression of *Dlx5* and *Hand1/2* in the MA it is not surprising that we observed an expansion of *Dlx5* and *Barx1* medio-distally in *Meis2* mutants. *Dlx5/6* are inhibited in medial–distal MA by *Hand2* because Wnt1-Cre;*Hand2*^f/f^ conditional mutants show increased *Dlx5/6* expression and decreased *Hand1* (Barron et al., 2011; Vincentz et al., 2016). It is noteworthy that *Dlx5/6* mutants do not exhibit aglossia, which is unlike Shh pathway and *Hand2* mutants (Clouthier et al., 2010). Furthermore, the homeobox protein Gsc, which is normally expressed in the aboral region of the MA, is downregulated both in Shh^−/−^ and in Wnt1-Cre;*Hand2*^f/f^ mutants, indicating again a similar regulatory circuit of Meis2 and Shh signalling. (Barron et al., 2011; Yamagishi et al., 2006). Xu et al. (2019) reported that elimination of Shh pathway in the mandibular arch of Wnt1-Cre;*Smo*^c/c^ led to the expansion of Bmp signalling activity through the oral–aboral axis which ultimately resulted in a mirror-image duplication of the mandibular bone. However, we did not detect elevated expression of *Bmp4* mRNA and Bmp targets such as Msx2 in *Meis2* mutants, nor did we see a duplication of the mandibular bone. Unexpectedly, we observed decreased expression of *Msx2*, indicating Bmp-independent mechanism of *Msx2* regulation. A residual Shh activity may operate in the MA mesenchyme that could be reflected by incomplete elimination of Hand2 in *Meis2* mutants. This may be sufficient for controlling physiological levels of Bmp activity. In sum, our data show that Meis2 is a key player in the gene regulatory network that includes temporospatial Shh and Bmp activity, and Hand, Dlx and Msx transcription machinery. Based on our data, Meis2 does not seem to be crucial for the fusion of lingual swellings at initial stages, but rather for subsequent growth of the tongue primordium, which is critically dependent on Shh activity. Impairment of tongue growth may result from the improper specification of NCC in the tongue primordium that we documented by ectopic ossification in this region.

### Mandibular ossification

Wnt1-Cre2;*Meis2*^fl/fl^ mutants display ectopic ossification of the MA tissue, i.e. broadened alveolar ridges and the presence of osteoblast-like cells close to lingual grooves. This is reflected by increased expression of *Runx2*, *Sp7* and ALP at more advanced stages. Furthermore, *Meis2* mutants exhibit fusion of the distal mandible that includes ossification of the distal tip and loss of incisors. This fusion is reminiscent of abnormal mandibles in Wnt1-Cre;*Hand2*^f/f^ embryos (Barron et al., 2011) and indicates a close molecular interaction of Shh pathway, Meis2 and Hand1/2 in developing the MA. It has been reported that Hand2 inhibits Runx2 transcriptional activity either by binding directly to Runx2 protein or by inhibiting expression of *Dlx5/6* in the medial–distal tip (Barron et al., 2011; Funato et al., 2009). However, it is important to note that Dlx5 affects *Runx2* expression and thus spatial shifts of Dlx5 in the absence of Meis2 may trigger abnormal ossification (Robledo et al., 2002; Samee et al., 2008). Alternatively, Meis2 may regulate Runx2 expression through the Dlx5/6-Hand2 circuit that is dependent primarily on the Shh activity.

### Muscle and tendon formation in the tongue

NCCs, epithelium and myogenic progenitor cells within the developing tongue share an intricate network of signalling interactions. It has been suggested that the neural crest acts as a scaffold for the organisation of migrating myogenic progenitor into the mesenchymal core of the arch, while simultaneously releasing molecules that instruct survival, proliferation and differentiation of myogenic progenitor cells as well as patterning of musculature (Parada and Chai, 2015; Parada et al., 2012). Mesodermally-derived myogenic progenitor cells migrate out from occipital somites and travel along a hypoglossal cord (mesodermal outgrowth from the anterior-most occipital somites) until they finally reach the newly formed tongue primordium (Harel et al., 2009). These cells express Pax3, which controls the differentiation of somitic mesoderm and skeletal muscle (Tajbakhsh and Cossu, 1997). As such, reduced levels of the *Pax3* gene result in disorganisation and deficiency of musculature (Zhou et al., 2008). In Wnt1-Cre2;*Meis2*^fl/fl^ mouse mutants we observed disrupted arrangement of both intrinsic (e.g. musculus longitudinalis superior) and extrinsic (e.g. genioglossus) musculature. Our findings are again similar to mouse mutants in which Shh activity is decreased. Shh directly influences the formation of NCC-derived lingual septum and aponeurosis, a fibrous band to which both intrinsic and extrinsic tongue muscles attach, and therefore is required for normal arrangement of musculature (Okuhara et al., 2019).

*Meis2* mutants express low levels of Sox9 in the tongue, which controls differentiation of NCC-derived tendons. Downregulation of Sox9 in the tongue results in failure of proper anchorage of muscles. *Meis2* mutants essentially phenocopy Sox9 pattern in Shh^MFCS4/−^ mutants (Okuhara et al., 2019), which lack Shh enhancer driving Shh expression in the oral ectoderm. However, Meis2 cannot directly control ectodermal Shh enhancer MFCS4 because its expression in the oral epithelium is not affected in Wnt1-Cre2;*Meis2*^fl/fl^ mutants. We speculate that Shh is affected by a cell non-autonomous mechanism from the adjacent mesenchyme that may involve Fgf signalling (e.g. Fgf10) (Lan and Jiang, 2009; Rice et al., 2004; Yamagishi et al., 2006).

Some ciliopathic mutants exhibit craniofacial anomalies similar to Shh pathway mutants. Kif3a is a protein residing in the primary cilium and is responsible for moving molecular cargo towards the plus end of microtubule. Loss of Kif3a in NCCs abrogates ciliogenesis and therefore blocks Shh signal transduction required for posttranslational modification of Gli proteins. Complete aglossia in Wnt1-Cre;*Kif3a*^f/f^ mutants is caused by a failure of invasion of mesoderm into the neural-crest derived mesenchyme of the tongue primordium. Increased apoptosis of NCCs and myogenic progenitors in the mandibular arch certainly plays a role in the origin of aglossia in *Kif3a* mutants (Millington et al., 2017). Both in *Kif3a* and in *Meis2* mutants, the tongue primordium does not bulge out from the mandibular arch and due to the failure in cell specification it probably lacks signals, which are necessary for invasion of myogenic progenitors.

A hallmark of improper NCCs specification might be the decreased expression of *Pax3* that is seen in Meis2 mutants. Pax3 is a transcription factor that is expressed in the neural-crest derived mesenchyme of tongue and mandible where it possibly keeps mesenchymal cells in an undifferentiated state (Wu et al., 2008). However, its role as a master regulator of neural-crest derived mesenchyme differentiation is poorly understood (Wu et al., 2008). *Pax3* is robustly expressed in cranial NCCs that make up the entire palatal, lingual and mandibular mesenchyme. Later on, the mesenchymal expression localises to the distal tip of tongue and the mandible. *Pax3* mutants with persistent *Pax3* overexpression in the entire mandibular arch, including the tongue, display defects in osteogenesis. Pax3 secretes a soluble inhibitor Sotdc1, which diminishes responsiveness to BMP and decreases expression of *Runx2* (Wu et al., 2008). We see similar molecular changes in Wnt1-Cre2;*Meis2*^fl/fl^ mutants with reduced Pax3 expression in the tongue primordium and increased Runx2 in comparison with control littermates.

In particular, *Pax3* expression is almost lost around the lingual groove, an epithelial invagination that forms a space that eventually separates the tongue from the alveolar bone. In the lingual groove, submandibular and sublingual ducts invaginate and branch to form mature glands of epithelial origin. In *Meis2* mutants, lingual grooves are extremely shallow and do not invaginate to create proper separation of the tongue and future mandibular bone. This malformed tissue ectopically expresses *Runx2* instead of *Pax3* indicating that the mesenchyme surrounding the lingual groove is not properly specified and rather adopts osteoblast-lineage fate. Indeed, we observe broader alveolar ridges and ectopic ossification around the lingual groove reaching medially towards the rudimentary tongue. Thus, failed medio-lateral patterning in *Meis2* mutants at E10.5 leads to abnormal differentiation of tongue-specific NCCs and hypoglossia.

### Cell proliferation and apoptosis

In many mouse mutants with eliminated Shh activity, loss of tongue and mandibular tissue were accompanied by increased cell apoptosis (Billmyre and Klingensmith, 2015; Millington et al., 2017; Okuhara et al., 2019; Xu et al., 2019; Yamagishi et al., 2006). As a whole, the cell proliferation index remained unchanged in Shh pathway mutants, although results were contradictory in one case. Wnt1-Cre;*Smo*^c/c^ mutants exhibited increased apoptosis and no change in cell proliferation in the MA, whereas the same mutant mice in another experiment displayed decreased cell proliferation along with increased apoptosis (Jeong et al., 2004).

Another aglossic mutant strain Wnt1-Cre;Hand2^f/f^ shows no major changes in proliferation, while apoptosis remains elevated (Barron et al., 2011). In Wnt1-Cre2;*Meis2*^fl/fl^ apoptosis is significantly increased while cell proliferation is normal, which is again in accordance with findings in Shh mutants. We observed elevated apoptosis mainly in the lateral regions of PA1 and to a lesser extent in the tongue primordium. Therefore, we assume that apoptosis represents a secondary effect and cannot explain hypoglossia as such. However, increased apoptosis in the MA may contribute to micrognathia, but micrognathia may also be a result of ectopic ossification. Similar lateral localisation of elevated apoptosis was observed in other studies in aglossic mutants. (Barron et al., 2011; Billmyre and Klingensmith, 2015).

## MATERIALS AND METHODS

### Mouse strains

Generation of the floxed allele of *Meis2* gene (*Meis2*^fl/fl^) with loxP sites around exons 2–6 was described in Machon et al. (2015). Conditional *Meis1*^fl/fl^ were generated from the embryonic stem cell clone HEPD0632_4_H07 purchased from EUCOMM. Frt-flanked LacZ/neo cassette was removed by ACTFLPe (strain #005703). LoxP sites flank exon ENSMUSE00000655363 encoding the homeobox region of the *Meis1* gene.

Wnt1–Cre2 mouse strain was purchased from The Jackson Laboratory (strain #022137) and it was used for specific deletion of *Meis1*^fl/fl^ or *Meis2*^fl/fl^ genes in neural crest cells. Reporter line mTmG was purchased from The Jackson Laboratory (strain #007676).

All procedures involving experimental animals were approved by the Institutional Committee for Animal Care and Use (permission #PP-084/2014). This work did not include human subjects.

### Immunohistochemistry

Embryos were fixed in 4% paraformaldehyde overnight at 4°C. 8–10 μm cryosections or 5-μm (paraffin-embedded) sections were permeabilised in 0.1% Triton X-100 in PBS (PBT). Antigen retrieval was performed in 0.1 M citrate buffer under pressure boiling for 12 min. After blocking, sections were incubated overnight in a primary antibody (5% BSA in PBT), washed with PBS and incubated with a fluorescent secondary for 1 h. Nuclei were visualised by DAPI (4,6-diamidino-2-phenylindol, 0.1 μg ml^−1^, Roche). Primary antibodies: Meis2 (Novusbio H00004212-M01) 1:2000, Shh (Santa Cruz Biotechnology sc-9024) 1:2000, Goosecoid (Gsc) (R&D Systems AF4086) 1:1000, FoxF1 (R&D Systems AF4798) 1:1000, FoxF2 (R&D Systems AF6988) 1:1000, Pax3 (DSHB), Casp3 (Cell Signalling 9664) 1:1000, phospho-histone 3 (PH3) (Upstate 6-570) 1:2000, phosphoSmad1/5 (Thermo Fisher Scientific) 1:1000. Secondary antibodies: anti-mouse (-rat, -rabbit) Alexa Fluor488 or 594 (Life Technologies). Biotinylated-anti-mouse, -anti-rabbit, -anti-rat (Vector Laboratories), Vectastain ABC Elite kit and ImmPACT DAB substrate (all Vector Laboratories). Images were acquired in Leica MZ APO stereomicroscope with DC200 camera or Olympus SZX9 with DP72 camera. Fluorescence images were acquired in Zeiss AxioZoom V16 and Zeiss AxioObserver Z1 microscopes. Bright-field light images were acquired in Leica DMLB using Zeiss ZEN Blue software.

### Alcian Blue/Alizarin Red staining

Embryos at E16.5.5–17.5 were dissected and scalded in hot water (65–70°C, 2 min). They were dehydrated in 95% ethanol for 48–72 h, changing solution every 12 h. After Alcian Blue (Sigma-Aldrich) staining for 12 h, they were rinsed twice in ethanol and kept overnight. After clearing in 1% KOH for 2 h and they were stained with Alizarin Red (Sigma-Aldrich) for 5 h. Further clearing in 2% KOH was carried out overnight, then in glycerol (25%) and 2% KOH (75%) for 8 h and glycerol (50%) and 2% KOH (50%) for 48 h. Tissue sections were rehydrated and stained in 0.04% Alcian Blue solution for 10 min. Pictures were obtained using binocular microscope Olympus SYX9 and camera Olympus DP72.

### Mallory's trichrome staining

Paraffin sections were rehydrated and incubated in Bouin's solution for 2 h at 55°C. After washing in distilled water, sections were incubated in 0.5% acidic fuchsine for 2 min and in the solution 0.5% Aniline Blue/ 2% Orange G/ 1% phosphotungstic acid for 30 min. Slides were washed in 4% acetic acid, dehydrated and mounted.

### ALP staining

Frontal cryossections of embryonic heads were washed twice in ALP buffer (100 mM Tris-Cl pH 9.5, 100 mM NaCl, 50 mM MgCl_2_, 0.1% Tween-20) for 10 min. The staining was performed in the same buffer in the presence of NBT/BCIP substrate (Roche 11681451001) for 30 min.

### Microtomography

Embryos were fixed in 4% PFA for 2 days and soaked in Lugol's iodine for several days. Scanning was performed on the instrument Bruker Skyscan 1272 with the resolution 3 µm.

### Whole-mount *in situ* hybridization

Cloning of riboprobes. cDNAs were cloned into pGEM-T-easy vector (Promega) using primers:

*Barx1* forward CTGGAGTCCCCCACCAAGCC, reverse GAGGGGTAGAAGCCTCAGCG; *Dlx5* forward TAGACCAGAGCAGCTCCACA, reverse CTGTAGTCCCAAAACTGAGC; *Gsc* forward ATGCCCGCCAGCATGTTCAG, reverse GTCCTTGCGTCAGGCAAGCG; *Hand1* (kind gift from A. Firulli); *Hand2* forward CGGAAGGCGAGATGAGTCTG, reverse TCACTGCTTGAGCTCCAGGG; *Ptch1* fwd GACAAACTTTGACCCCTTGG, reverse GAAGACATCATCCACACCAA; *Msx1* forward CTGCATGGCCCCGGCTGCTG, reverse CTAAGTCAGGTGGTACATGC, (kind gift from V. Korinek); *Msx2* forward ATGGCTTCTCCGACTAAAGGC, reverse TTAGGATAGATGGTACATGC, (kind gift from V. Korinek); *Fgf8* forward CAGGTCCTGGCCAACAAG, reverse GAGCTCCCGCTGGATTCCT *Bmp4* (kind gift from B. Hogan).

Antisense mRNA was transcribed with T7 or SP6 polymerase. Whole-mount *in situ* hybridization was performed using standard protocols.

## Supplementary Material

Supplementary information
