# Supplementary material for: Neural crest cells require Meis2 for patterning the mandibular arch via the Sonic hedgehog pathway
Source: Biol Open. 2020 Jun 25;9(6):bio052043. doi: 10.1242/bio.052043 (PMC7331463; doi:10.1242/bio.052043)
Supplement: Supplementary information [file biolopen-9-052043-s1.pdf]

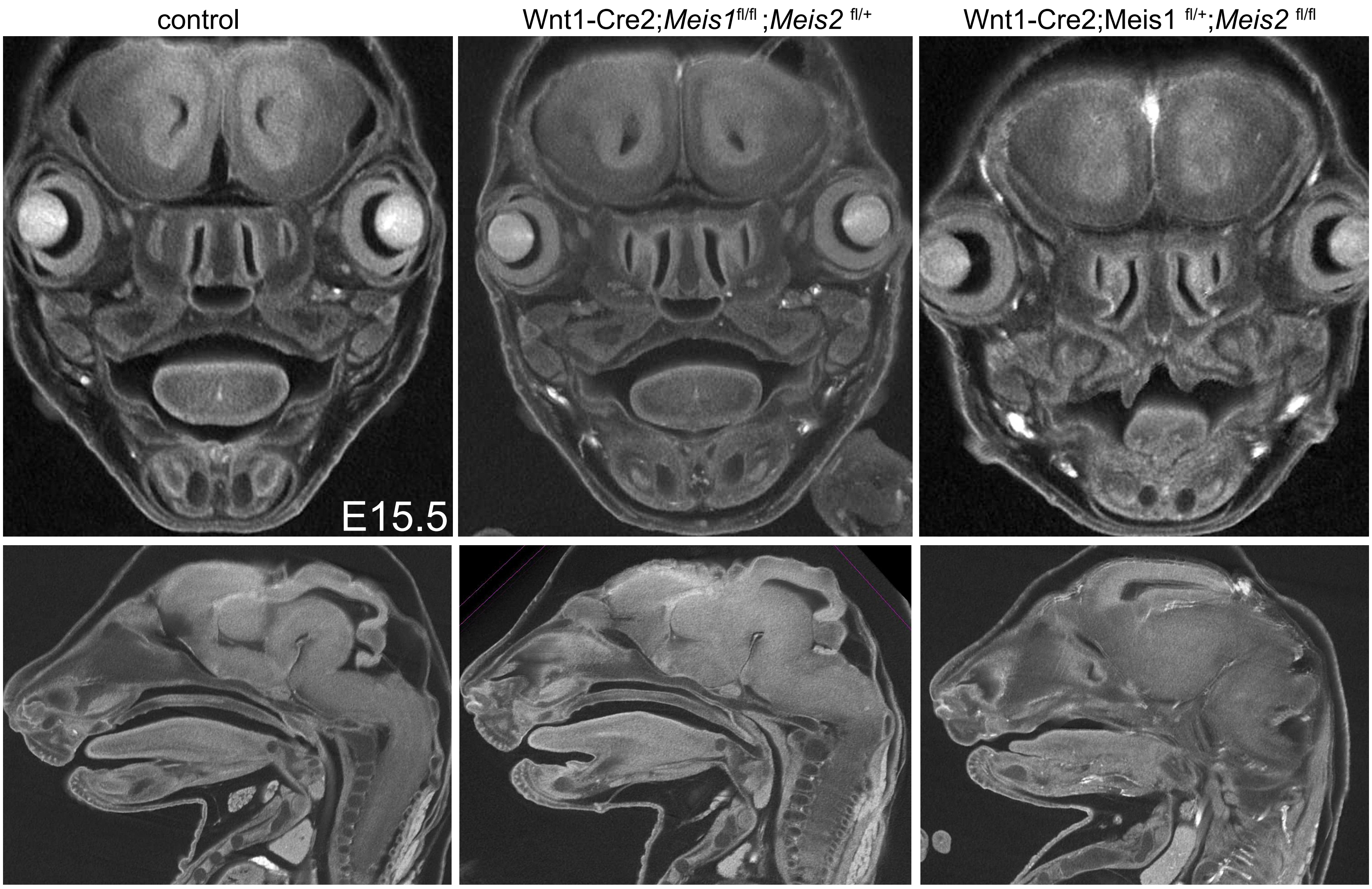

Fig. S1: micro-CT description of foeti at E15.5 from control littermates, Wnt1-Cre2;Meis1 fl/fl;Meis2 fl/+ , and Wnt1-Cre2;Meis1 fl/+;Meis2 fl/fl
